# Base editors in zebrafish: a new era for functional genomics and disease modeling

**DOI:** 10.3389/fgeed.2025.1598887

**Published:** 2025-05-21

**Authors:** Yuwen Liu, Chao Li, Yiren Qiu, Sihong Chen, Yijun Luo, Donghua Xiong, Jun Zhao, Jianmin Ye, Xuegeng Wang, Wei Qin, Fang Liang

**Affiliations:** ^1^ Guangzhou Key Laboratory of Subtropical Biodiversity and Biomonitoring, Guangdong Provincial Engineering Technology Research Center for Environmentally-friendly Aquaculture, Institute of Modern Aquaculture Science and Engineering, School of Life Sciences, South China Normal University, Guangzhou, Guangdong, China; ^2^ School of Life Sciences, Sun Yat-sen University, Guangzhou, Guangdong, China; ^3^ Genes and Human Disease Research Program, Oklahoma Medical Research Foundation, Oklahoma City, OK, United States

**Keywords:** CRISPR-Cas9, base editing, cytosine base editors, adenine base editors, zebrafish, disease modeling

## Abstract

Base editing has revolutionized genome engineering by enabling precise single-nucleotide modifications without inducing double-strand breaks. As a powerful and efficient gene-editing tool, base editors (BEs) have been widely applied in various model organisms, including zebrafish (*Danio rerio*), to facilitate functional genomic studies and disease modeling. Zebrafish, with its genetic similarity to humans and rapid development, provides an excellent platform for testing and optimizing emerging base editing technologies. This review comprehensively explores the advancements of cytosine and adenine base editors in zebrafish, highlighting recent developments that enhance efficiency, specificity, and editing scope. We discuss novel base editor variants tailored for zebrafish applications, improvements in delivery strategies, and methodologies to minimize off-target effects. Furthermore, we compare base editing with other precision genome-editing technologies, such as prime editing and homology-directed repair, to underscore its advantages in achieving targeted mutations with high fidelity. By evaluating the expanding role of base editing in zebrafish, this review provides valuable insights into its potential for translational research, genetic disease modeling, and future therapeutic applications.

## 1 Introduction

Zebrafish (*Danio rerio*) have emerged as a pivotal model organism for single nucleotide editing due to their genetic similarity to humans, transparent embryos, and rapid development (Howe et al., 2013; Uribe-Salazar et al., 2022). Among the various gene editing technologies, base editors (BEs) have shown significant promise for introducing precise single-nucleotide changes without inducing double-strand breaks (DSBs). However, the application of BEs in zebrafish presents unique challenges, including bystander mutations from wide activity windows, off-target effects, and the need for efficient delivery methods such as microinjection, electroporation, and transduction (Rees et al., 2017; Zhang et al., 2017). This review aims to provide a comprehensive overview of the advancements, applications, and challenges of base editing technologies in zebrafish, with a particular focus on their use in disease modeling and functional genomics.

## 2 Molecular mechanisms of base editors

### 2.1 Principles and methodologies of base editing technologies

CRISPR-Cas9 is the most reliable and widely used editing tool in genetic research. Guided by gRNA, the nucleases introduce double-strand breaks (DSBs) at the target site of the specific DNA sequence. These DSBs activate the cell’s DNA-repair mechanisms, which include non-homologous end joining (NHEJ) and homology-directed repair (HDR). Since HDR methods are more precise as they utilize homologous DNA sequences, researchers inhibit NHEJ and stimulate HDR to enable precise genome editing (Feng et al., 2023). However, yielding efficient and stable single-nucleotide mutations is challenging with DSB-mediated HR. Gene editing methods that target sequences without inducing DSBs and that use DNA-free components are therefore increasingly preferred (Li Y. et al., 2021). The advent of base editing has generated great advancements, particularly with its unparalleled accuracy, which enables single-nucleotide level modifications.

**FIGURE 1:**
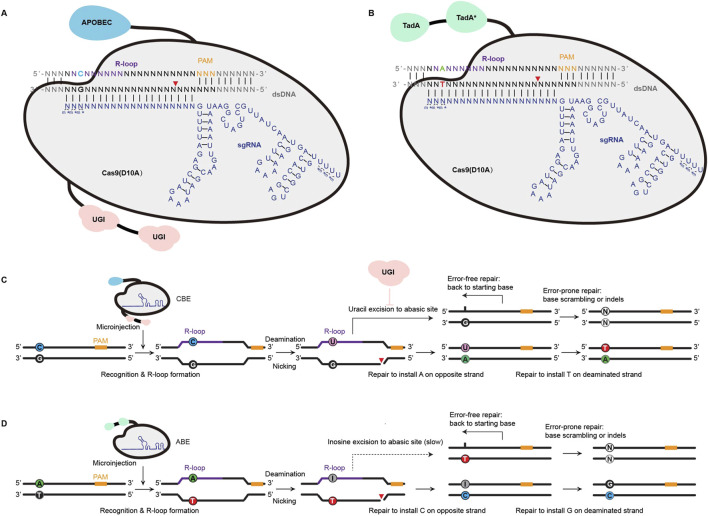
Principle of inducing single nucleotide mutations with Cas9 base editors (BEs). **(A)** CBE involves APOBEC (cytidine deaminase) and Cas9 (D10A) for precise C•G to T•A conversion. Guided by sgRNA, the system forms an R-loop in the target double-stranded DNA (dsDNA), exposing the non-complementary strand for cytosine deamination. UGI (uracil DNA glycosylase inhibitor) prevents uracil removal, allowing repair to result in targeted base substitution. **(B)** ABE, which involves TadA (adenine deaminase) and a Cas9 (D10A)-sgRNA complex, facilitates A•T to G•C base conversion through targeted adenine deamination, producing inosine, which is subsequently repaired as guanine. SgRNA with chemical modifications comprising 2-0-Methyl analog at the first three and last four bases and 3′phosphorothioate bonds between three first and last bases loaded into Cas9 protein is symbolized with m*. **(C,D)** illustrate the repair mechanisms of CBE and ABE, respectively. For CBE, cytosine deamination generates uracil, followed by nicking and repair, leading to C-to-T or G-to-A transitions on the non-edited strand. Error-free repair restores the original base, while error-prone repair may introduce indels or other mismatches. For ABE, adenine deamination produces inosine, which slowly converts to guanine during repair. Similarly, ABE generates A-to-G or T-to-C transitions with potential error-prone repair outcomes. These systems enable precise and programmable single-base modifications, critical for targeted genome editing applications.

Base editing enables the direct conversion of one nucleotide into another without creating double-strand breaks. This technology mainly contains cytosine base editors (CBEs) and adenine base editors (ABEs), which utilize engineered enzymes like cytosine or adenine deaminases (Komor et al., 2016; Gaudelli et al., 2017). CBEs are the first technology to bypass the need for endogenous DSB repair, enabling precise C:G to T:A conversions. CBEs achieve this by fusing a catalytically inactive Cas nuclease domain (e.g., Cas9 nickase or dCas9)—which cannot induce DSBs—to the APOBEC1 (apolipoprotein B mRNA-editing enzyme catalytic subunit 1) cytidine deaminase and uracil glycosylase inhibitor (UGI) domain (Komor et al., 2016) (Figure 1A). When the single-guide RNA (sgRNA)–CBE complex binds to its target sequence, the guide RNA spacer hybridizes to the target DNA strand, causing displacement of the PAM-containing genomic DNA strand. This results in a ssDNA R-loop, which determines the location of the editing window. Then, the APOBEC1 cytidine deaminases convert all cytosines within the editting window into uracils, which DNA polymerases then interpret as thymines (Anzalone et al., 2020). As the deaminase operates on single-stranded DNA, the sgRNA is designed to bind the target strand of the target locus, which contains the C:G base pair to be edited. The Cas9 nickase component cuts the non-target strand, which triggers DNA repair and increases the conversion of U:G base pairs into T:A pairs (Figure 1C). Similarly, ABEs catalyze A:T to G:C conversions by using an adenine deaminase instead of APOBEC1. This converts adenosines within the R-loop to inosines, which are then recognized as guanines by DNA polymerases (Gaudelli et al., 2017) (Figures 1B,D).

### 2.2 The development of base editors in zebrafish

**FIGURE 2:**
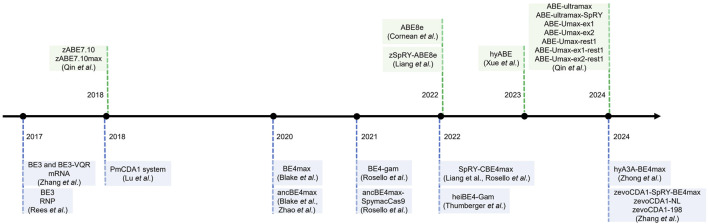
The timeline of base editing development in zebrafish.

Although various single-nucleotide editors have been developed, extending their applications to zebrafish has been slow (Figure 2). In 2017, David Liu’s group proposed a high-fidelity version of BE3 by inducing four-point mutations (N497A, R661A, Q695A and Q926A). The engineered system HF-BE3 retains the basic functionalities of BE3, showing comparable *in vitro* editing efficiency and activity window withs while exhibiting higher editing specificity and lower off-target rates—reducing off-target effect by 37-fold at non-repetitive sites and by 3-fold at a highly repetitive site (Rees et al., 2017). In the same year, W. Qin and H.A. Rees et al. pioneered using the cytosine base editor, BE3, in zebrafish by microinjecting mRNA or ribonucleoprotein (RNP) complexes. With this, they achieved editing efficiencies ranging between 9.25% and 28.57% and successfully established an oculocutaneous albinism (OCA) disease model. Furthermore, they used artificial Cas9 variants nCas9 and dCas9 to create a BE-VQR and dCas9-vqr fusion protein, respectively resulting in the recognition of 5′-NGA PAM and a reduction in the indel formation rate (Rees et al., 2017; Zhang et al., 2017; Qin et al., 2018a). Following this, X. Lu et al. introduced a codon-optimized Target-AID system for zebrafish, which is based on the PmCDA1 deaminase, featuring a unique editing window targeting −19 to −16 nucleotides upstream of the PAM sequence. This specific targeting range made Target-AID complementary to other base editors (Qin et al., 2018a; Lu et al., 2018). In 2020, Y. Zhao and B. Carrington et al. associated Anc689 with BE4max, modified the AncBE4max nucleotide sequence to be compatible with zebrafish codons, GC content, and secondary structures, then realized the application of AncBE4max system in zebrafish, which enhanced editing efficiency by approximately threefold compared with the BE3 system (Zhao et al., 2020; Carrington et al., 2020). Around the same time, M. Rosello et al. demonstrated the effectiveness of the BE4-gam and AncBE4max base editing systems in zebrafish by successfully inducing oncogenic mutations in tumor suppressor genes, such as tp53, and showcasing their utility in cancer modeling. Compared with the classic BE4-gam, ancBE4max shows astonishing progress with increased efficiency by 90%. Replacing the PIM domain of SpCas9 in ancBE4max with that of SpymacCas9, Spymac-ancBE4max has a PAM preference for NAA, which significantly expands the effect scope of the base editor (Rosello et al., 2021). In 2023, F. Liang and M. Rosello et al. reported the development of a “near PAM-less” cytidine base editor (CBE4max-SpRY) for zebrafish, which bypasses the typical NGG PAM requirements inherent to other CRISPR-Cas9 systems. This tool can target virtually all PAM sequences and achieves exceptional base editing efficiencies, with some loci reaching rates of up to 87% (Liang et al., 2022; Rosello et al., 2022). That same year, A. Cornean et al. launched ACEofBASEs, an online platform that enables efficient sgRNA design and off-target prediction. In this study, researchers utilized the AncBE4max and EvoBE4max systems in medaka to precisely introduce stop-gain and missense variants in the *OCA2* gene (associated with eye pigmentation) and the *KCNH6A* gene (associated with potassium channels). They also used these tools to validate four congenital heart disease (CHD) candidate genes (*DAPK3, UBE2B, USP44,* and *PTPN11)*. This underscores the potential of these tools for rapid genotype-phenotype studies in F0 embryos, with subsequent confirmation of results in F1 generations (Cornean et al., 2022). To further enhance performance, Thumberger et al. (2022) incorporated a “hei-tag” (high-efficiency tag) into base editors by combining a Myc tag coupled to an optimized nuclear localization signal (NLS). The hei-tag improves the efficiency of gene editing by immediately shuttling the Cas9 enzyme into the nuclear compartment where the DNA is stored, while the ORF of BE4-Gam expanded with hei-tag significantly improves the efficiency of genome targeting. With heiBE4-Gam, all C-to-T transitions at the OlOca2 T1 target site are increased by approximately 1.7-fold (Thumberger et al., 2022). In 2024, Z. Zhong et al. advanced precision genome editing in zebrafish by integrating Rad51 DNA-binding domains into single-nucleotide editors, such as hyA3A-BE4max. They developed an improved variant, zhyA3A-CBE5, that not only increased editing efficiency but also extended the editing window from C3–C11 to C3–C16 near the PAM site. Moreover, Cas-OFFinder and high-throughput sequencing (HTS) analysis showed that the off-target editing of zhyA3A-CBE5 was almost imperceptible (Zhong et al., 2024). In parallel, Zhang et al. engineered zevoCDA1, a cytosine base editor optimized for zebrafish, that enhanced editing performance across diverse DNA contexts and reduced PAM sequence constraints. Additionally, Y. Zhang et al. developed zevoCDA1-198, a more precise cytosine base editor with a focused editing window spanning just five nucleotides that substantially reduced off-target effects—as virtually no non-target edits detected when creating disease models (Zhang et al., 2024). These tools led to the creation of zebrafish disease models that were previously challenging to generate due to sequence-related limitations.

Adenine base editors (ABEs) have been less extensively studied in zebrafish than cytosine base editors (CBEs). In 2018, W. Qin et al. introduced zABE7.10max—an ABE system that enabled functional adenine base editing in zebrafish by utilizing codon optimization and replacing NLS signal peptides. It is proved that ABE7.10 has a very high product purity (typically >99.9%), however, its limited success across various target loci hindered its broader applicability (Qin et al., 2018b). Following this, A. Cornean and F. Liang et al. demonstrated the utility of ABE8e and its variant, ABE8e-SpRY, in zebrafish by disrupting the *TSR2* gene to construct a disease model of Diamond-Blackfan anemia that mimicked its morphological defects. This highlighted zSpRY-ABE8e as an efficient and precise gene editing tool for functional genetic studies and disease modeling in zebrafish (Liang et al., 2022; Cornean et al., 2022). In 2023, N. Xue et al. developed a hyperactive adenine base editor (hyABE) by fusing ABE8e with Rad51DBD. This significantly improved the efficiency of A-to-G conversions near the PAM region by up to 7-fold compared with ABE8e (Xue et al., 2023). In 2024, W. Qin et al. constructed a new ABE system, ABE-Ultramax (ABE-Umax), through *in vivo* model screening in zebrafish, achieving an editing efficiency of up to 86%—a 3-fold improvement over ABE8e. Building on this, Qin et al. developed two novel variants, ABE-Umax-ex1 and ABE-Umax-ex2, featuring expanded and shifted editing windows to target a broader range of genomic loci. Additionally, they also developed a precision tool, ABE-Umax-rest1, that enhances editing accuracy by considerably reducing bystander mutations. The successful construction of multiple zebrafish disease models, including models with missense and splicing mutations, further validated the applicability and robustness of this tool. This breakthrough enables single-nucleotide variants (SNVs) to be functionally validated and disease models to be established in zebrafish, providing a versatile and powerful toolkit for genetic research with further potential applications in gene therapy (Qin et al., 2024). The SpRY-CBE4max, SpRY-zABE8e, and ABE-Umax suite of base editors increase sequence requirement flexibility and have lower frequencies of indels, overcoming the considerable limitation of PAM-dependent base editing windows (Liang et al., 2022; Qin et al., 2024; Zheng et al., 2023).

In addition to conventional single-nucleotide editing of nuclear DNA, mitochondrial DNA base editing became a research hotspot. In 2019, Bian, W. P. et al. developed a promising mito-CRISPR/Cas9 system to edit mtDNA in zebrafish by knock-in strategy (Bian et al., 2019). To further achieve more precise editing in mitochondria, Stephen C Ekker and B. Shen et al. employed DddA_tox_-TALE strategy to modify the existing DdCBE, and selected a derivative editor with optimized performance (DddA_tox_ split at 1,397 amino acid position to obtain the L1397C + R1397N pair) to achieve efficient gene editing in zebrafish, with editing efficiency of 60%–85% at multiple sites in zebrafish mtDNA, and some reaching as high as 90% (Sabharwal et al., 2021; Guo et al., 2021). Their studies demonstrated the application of a double-stranded DNA cytosine base editor (DdCBE) for mitochondrial DNA (mtDNA) base editing in zebrafish, which establishes zebrafish as a valuable model for mitochondrial diseases, offering insights into their pathogenesis and potential therapeutic strategies.

From 2017 onwards, base editing technology in zebrafish has seen steady development, resulting in the establishment of various tools (Table. 1). These base editors display enhanced compatibility with zebrafish codons, achieving higher species-specific targeting efficiencies. Consequently, they present promising prospects for advancing zebrafish applications in disease modeling, drug discovery, and therapeutic development.

**TABLE 1:** The list of base editors used in zebrafish.

Base editor	Editing window	PAM	Type	Efficiency	References
SpCas9-BE3	C4 - C8	NGG	CBE	9.25% - 28.57%	(Qin et al., 2018a; Rees et al., 2017; Zhang et al., 2017)
Target-AID	C2 - C6	NGG	CBE	15.00%- 35.00%	(Lu et al., 2018)
zancBE4max	C4 - C8	NGG	CBE	21.77% - 67.36%	(Zhao et al., 2020)
BE4-gam	NA	NGG	CBE	62.50% ^**a**^	(Rosello et al., 2021)
ancBE4max-SpymacCas9	NA	NAA	CBE	19.00% ^**a**^	(Rosello et al., 2021)
SpRY-CBE4max	C4 - C9	NNN, NRN > NYN	CBE	100.00% ^**a**^	(Liang et al., 2022; Rosello et al., 2022)
heiBE4-Gam	NA	NGG	CBE	74.10% ± 8.90% ^**a**^	(Thumberger et al., 2022)
hyA3A-BE4max	C3 - C16	NGG	CBE	18.86% - 62.30%	(Zhong et al., 2024)
zevoCDA1-SpRY-BE4max	C1 - C10	NNN, NRN > NYN	CBE	25.00% - 90.00%	(Zhang et al., 2024)
zevoCDA1-NL	C1 - C7	NNN, NRN > NYN	CBE	78% ^**a**^	(Zhang et al., 2024)
zevoCDA1-198	C1 - C6	NNN, NRN > NYN	CBE	79% ^**a**^	(Zhang et al., 2024)
zABE7.10	A5 - A7	NGG	ABE	7.14% - 22.20%	(Qin et al., 2018b)
zABE7.10max	A5 - A7	NGG	ABE	19.20% - 40.70%	(Qin et al., 2018b)
ABE8e	A4 - A8	NGG	ABE	92.90% ± 3.70% ^**a**^	(Cornean et al., 2022)
zSpRY-ABE8e	A3 - A9	NNN, NRN > NYN	ABE	98% ^**a**^	(Liang et al., 2022)
hyABE	A2 - A15	NGG	ABE	68% ^**a**^	(Xue et al., 2023)
ABE-ultramax	A4 - A8	NGG	ABE	100% ^**a**^	(Qin et al., 2024)
ABE-ultramax-SpRY	A4 - A8	NNN, NRN > NYN	ABE	88% ^**a**^	(Qin et al., 2024)
ABE-Umax-ex1	A4 - A12	NGG	ABE	87% ^**a**^	(Qin et al., 2024)
ABE-Umax-ex2	A5 - A16	NGG	ABE	70% ^**a**^	(Qin et al., 2024)
ABE-Umax-rest1	A5 - A6	NGG	ABE	55% ^**a**^	(Qin et al., 2024)
ABE-Umax-ex1-rest1	A4 - A6	NGG	ABE	9.50% - 60.50%	(Qin et al., 2024)
ABE-Umax-ex2-rest1	A12 - A15	NGG	ABE	23.00% - 68.50%	(Qin et al., 2024)

a, highest efficiency reached.

### 2.3 Delivery methods for base editing in zebrafish

**FIGURE 3:**
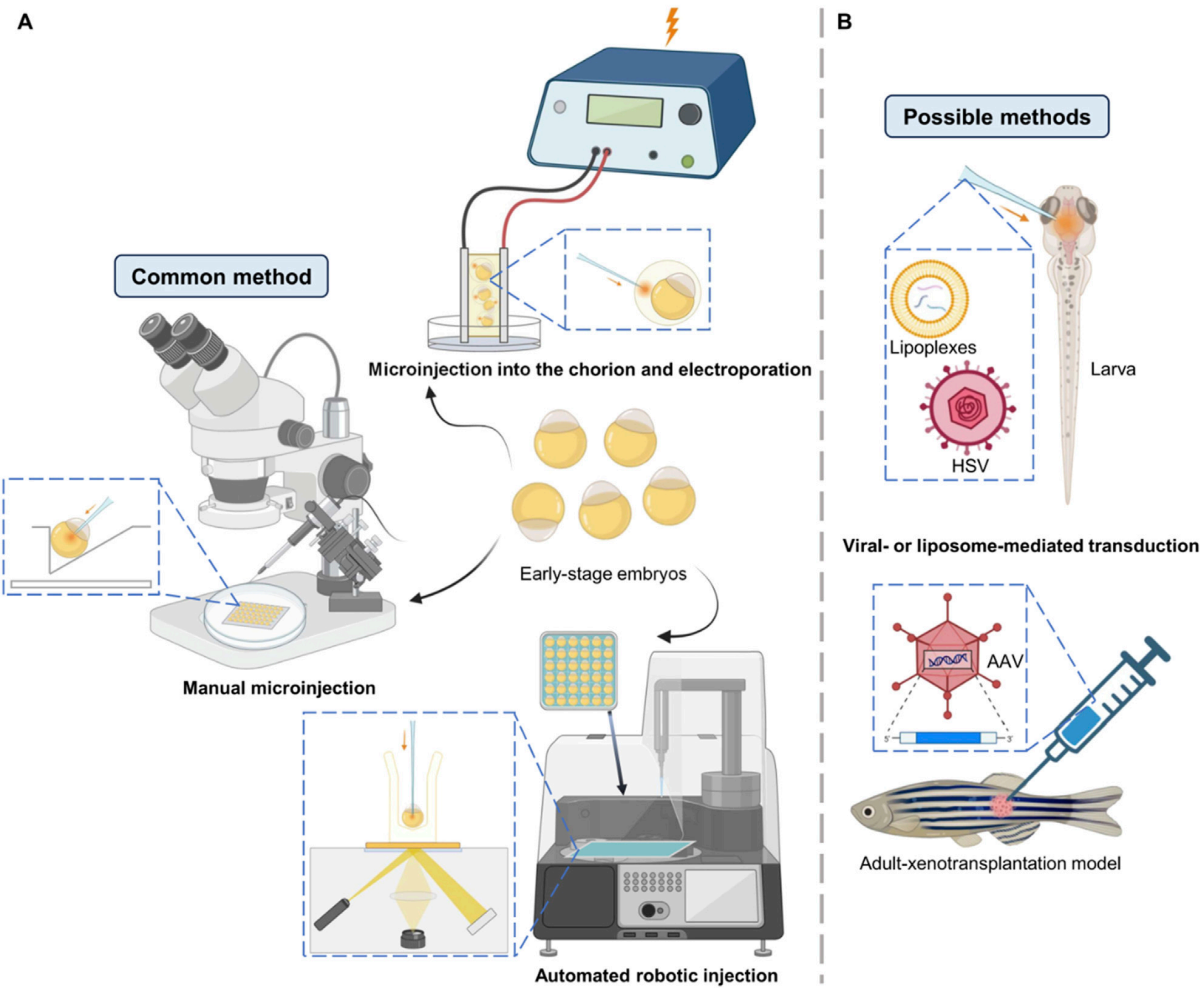
Delivery methods illustration in zebrafish. **(A)** Common methods to deliver mRNA, RNP or plasmid into early-stage embryos. Manual microinjection is the mostly widely used method for base editing in zebrafish. **(B)** Some other possible methods to deliver nucleic acids in larvae or adults.

The efficiency of base editing in zebrafish heavily relies on the delivery method used to introduce the editing components into the embryos. The most common methods include microinjection, electroporation, and viral transduction (Figure 3). Microinjection is the most widely used technique due to its high precision and ability to deliver RNP complexes directly into zebrafish embryos at the one-cell stage (Rees et al., 2017; Liang et al., 2022; Liu et al., 2019). However, most of the base editor delivery in zebrafish is by injection of a mixture of mRNA and gRNA rather than RNP (Zhang et al., 2017; Lu et al., 2018; Zhao et al., 2020; Qin et al., 2018b). Manual microinjection is prevalent but laborious and time-consuming, and its efficiency can vary depending on the skill of the operator, whereas automated robotic injection come into sight since it has no flaws as above, which is proved to be highly suitable as a high-throughput alternative to manual injection (Del Prado et al., 2024; Guo et al., 2025). To further improve the effectiveness and survival of injections, researchers have made improvements in both experimental techniques and instruments, for example, 0.25% trypsin is used to debind zebrafish embryonic mucus to reduce obstruction to needle entry and drug injection, and needles are modified to make them more penetrating and effective in preventing reflux (Lu et al., 2021).

Unlike microinjection, which is used for early embryos, electroporation is mostly used to deliver editing components into later-stage embryos or tissues, allowing precise editing to particular cells in zebrafish at specific developmental stages (Wang et al., 2024; Yao et al., 2024). These advantages have made it favored for the construction of zebrafish cancer models—the technique of modeling melanoma in adult zebrafish using electroporation is relatively well established, allowing for spatial and temporal control of tumorigenesis (Callahan et al., 2018; Mito et al., 2022). However, electroporation may cause cell damage and has lower editing efficiency compared to microinjection, which limits its wide application in mutating embryos. In order to explore new possibilities for gene delivery in zebrafish embryos, Tazin et al. developed a novel method by combining microinjection into the space between the chorion and the embryo followed by electroporation, which demonstrates the prospect of electroporation for the delivery of base editors (Tazin et al., 2023).

Viral transduction, particularly using adeno-associated viruses (AAVs), has been used in drug research and gene delivery in zebrafish (Khan et al., 2019a; Khan et al., 2019b; Satou et al., 2022), showing promise for delivering base editors *in vivo*, but its application is limited by the small size of the embryos and the potential for immune responses. Recently, liposome delivery has also received extensive attention. H. Zhang et al. proposed mRNA co-encapsulation in lipoplexes—mingle-lipoplexes containing defined ratios of two mRNAs result in more precise co-transfection of cells *in vivo* in zebrafish embryos, outperforming single-lipoplexes (Zhang et al., 2022). For base editing, liposome-mediated delivery of the RNP complex of BE3 directly into mammalian cells has been realized, so it is a reasonable prospect for this to be realized in zebrafish in the future (Rees et al., 2017). Each delivery method has its advantages and limitations, and the choice of method depends on the specific experimental requirements and the stage of zebrafish development.

The above methods mainly rely on embryo-mediated genome editing, so they often produce chimeras with different cells carrying different genotypes at the F0 generation, which will affect the accuracy of phenotypic determination. However, if the editing efficiency of the F0 generation is very high, even the chimera can be phenotyped in the F0 generation, thus shortening the experimental timeline. The high germline transmissibility rate reflects the high editing efficiency to a certain extent, and the zABE7.10 with a high germline transmissibility rate has been reported to randomly select positive individuals among the five targets of F0 zebrafish, and its germline transmissibility rate can reach 25%–58% (Qin et al., 2018b). The germline propagation efficiency of ABE Umax on the eight targets was more than 50%, and the optimal target even reached 80%–100% (Qin et al., 2024). If the editing efficiency of the F0 generation is high enough (the propagation rate of the F1 generation germline of ABE-Umax can reach 50.6% or more), the target phenotype can be directly identified at the F0 generation by PCR or genotype analysis. Conversely, if editing efficiency is low (<50%), a more conservative strategy is to outcross F0 fish with wild-type and evaluate stable homozygous lines in F1 (Qin et al., 2024). Given the short generation time of zebrafish, the method of obtaining stable fish lines by F1 generation is also widely adopted. The above two methods can be flexibly selected to deal with different scenarios.

## 3 Applications in base editing in zebrafish

### 3.1 Disease modelling with single-nucleotide mutations

Disease models that closely mimic human conditions can greatly enhance studies into disease pathogenesis and the development of drugs and treatments (Hanot et al., 2023; Lu et al., 2020). Zebrafish, as disease models, offer unique advantages, including their *ex vivo* early embryonic development, enabling gene knockouts that would otherwise be lethal in mice (Nanjappa et al., 2023; Jaako et al., 2011). Furthermore, disease simulation in zebrafish is very similar to that in mice, highlighting zebrafish as a reliable disease model (Dalla Barba et al., 2023; Ikle et al., 2022).

Gene knockouts have been a key method of establishing disease models and studying the role of specific genes. Over the past few decades, human diseases caused by single-nucleotide mutations have been modeled in zebrafish by either knocking out the disease gene, injecting point-mutated mRNA for transient expression, or knocking in a point-mutated disease gene (Hong et al., 2016; Livne et al., 2022). Before base editing, CRISPR/Cas technology disrupted gene function by knocking in large sections of genetic material, showing great promise for studying of single-nucleotide mutations (Zu et al., 2013; Bai et al., 2020; Li et al., 2019; Han et al., 2021). However, this approach was associated with several problems, particularly unpredictable DNA repair outcomes. In contrast, base editors reproduce the subtle changes in protein function caused by a single-nucleotide substitution, producing more precise and efficient results (Rosello et al., 2023).

**FIGURE 4:**
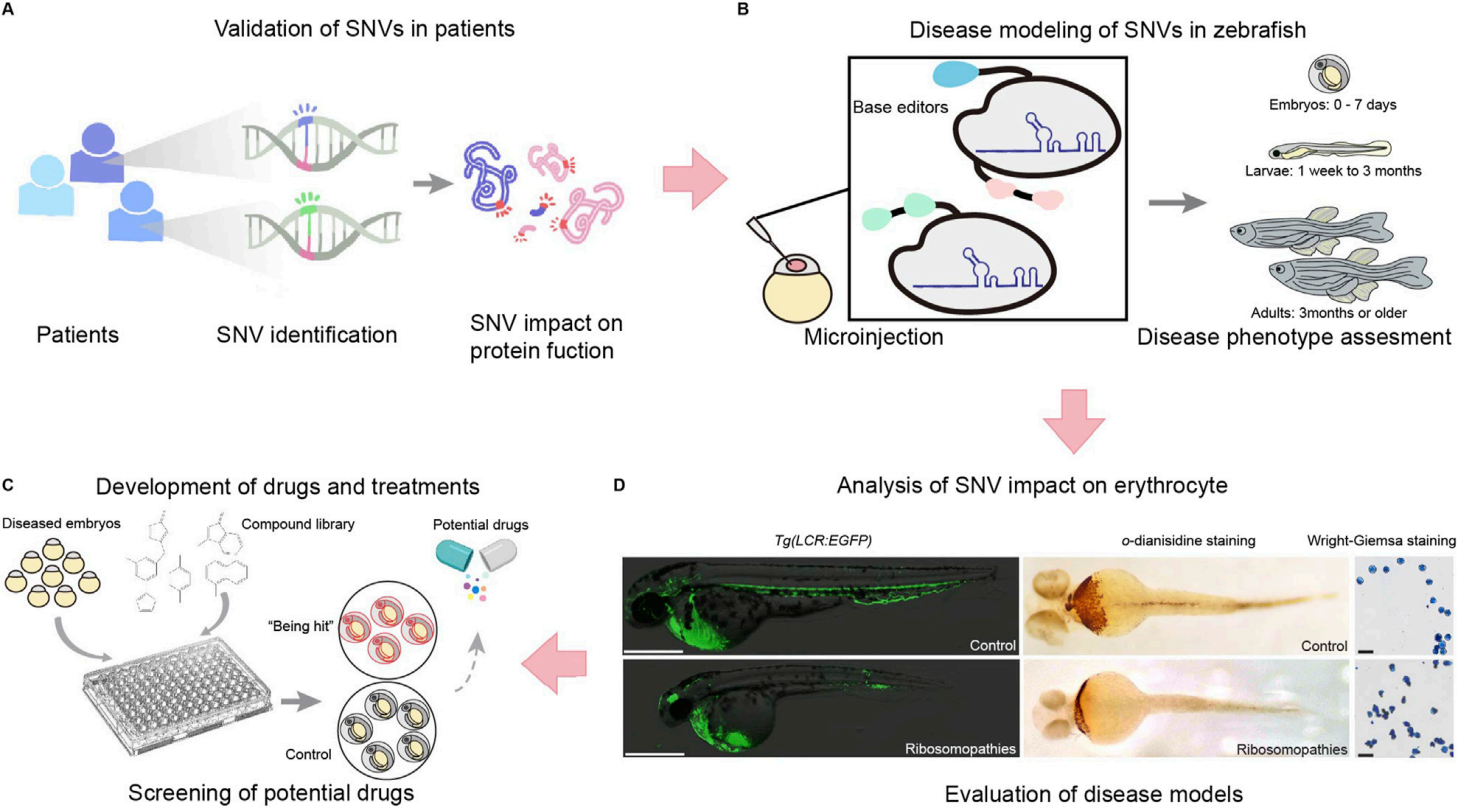
The application of single nucleotide editing in studying single nucleotide variations (SNVs), including validation, disease modeling, drug development, and functional analysis. **(A)** SNVs identified from patient samples are first assessed for their impact on protein function. **(B)** To further validate their role in disease, zebrafish models are generated using base editors and microinjection techniques to introduce specific SNVs into zebrafish embryos and evaluate disease phenotypes across developmental stages, from embryos (0–7 days) to larvae (1 week–3 months) and adults (3 months or older). **(C)** This system facilitates phenotypic assessments and provides a platform for drug screening. Diseased embryos are exposed to compound libraries, and potential therapeutic candidates are identified by comparing treated embryos to controls. **(D)** The impact of ribosomopathies related SNVs on erythrocytes is analyzed using erythrocyte specific transgenic zebrafish lines *Tg(LCR:EGFP)*, o-diansidine staining or Wright-Giemsa staining to detect hemoglobin and evaluate erythrocyte morphology. This integrated approach enables the evaluation of SNV-driven disease models and the identification of potential therapeutic strategies.

With these advancements, disease models can be rapidly created, enabling personalized drug screening (Rosello et al., 2022; Tessadori et al., 2018). Currently, several single-nucleotide mutation disease models have been created using base editing in zebrafish (Figures 4B,C). These include zebrafish models for dwarfism (Rosello et al., 2021), Ablepharon macrostomia syndrome (Zhao et al., 2020), Diamond-Blackfan anemia (Liang et al., 2022), and prelingual hearing loss (Qin et al., 2024).

### 3.2 Studying likely pathogenic single-nucleotide variants (SNVs)

Zebrafish disease models created using base editing technologies have enabled studies into the molecular basis of disease and the identification of therapeutic targets (Dark et al., 2020; Kolvenbach et al., 2023). In addition, studying and identifying potential disease candidate variants can provide insight into the prevention and treatment of genetic diseases (Blombery et al., 2023; Waters et al., 2024) (Figures 4A–D). However, individuals carrying mutations often have reduced fertility, which can complicate genetic investigations (Seda et al., 2023). To solve this problem, Qin et al. (2024) fused the 3′UTR of the *NANOS1* gene with the ABE-Umax base editor, resulting in a germline-specific base editor; this is a crucial method for high-throughput SNV phenotype screening.

### 3.3 Contributions to drug discovery and therapeutic development

Many genetic diseases exist, and while some have specific treatments, many do not, and sometimes patients develop drug resistance. Therefore, developing new drugs and therapies remains an urgent priority. To construct a zebrafish disease model, the corresponding homolog of the human pathogenic gene must first be identified. Mutants can then be created, enabling investigations into the gene’s pathogenicity and assessments of drug efficacy. Even if a cure cannot be identified, achieving a better understanding of the disease using zebrafish models could help to develop drugs to alleviate symptoms (He et al., 2021) (Figure 4C).

## 4 Challenges and future directions

### 4.1 Specificity

The single-base editor targeting window refers to the range of genomes in which a single-base editor can effectively catalyze base switching, which is usually determined by of the range of deaminase activity, the recognition site of the Cas protein, and the conformation of the linked peptide (Zhang et al., 2024). In zebrafish systems, the targeting window of the mainstream cytosine base editor (CBE) is usually located at C_4_∼C_8_ at the distal end of the PAM, such as BE4max; The targeting window of the adenine base editor (ABE) is concentrated in A_5_∼A_7_, such as ABE8e (Zheng et al., 2023; Kim et al., 2017). However, changing the type of deaminase, using a Cas variant, or designing a linker peptide may affect the width and location of the targeting window. For example, the introduction of Rad51 DBD can extend the targeting window of CBE to C_12_∼C_16_ proximal to PAM by enhancing the interaction between deaminase and DNA, while maintaining a 1.59∼3.50-fold increase in efficiency (Zhao et al., 2020). In zebrafish models, the targeting window is also affected by the type of diagnosis, etc., and the optimization of the targeting window is important for disease modeling. Traditional CBE, such as zAncBE4max, has a narrow targeting window of C_3_∼C_7_ in zebrafish, and the efficiency of GC/CC sequence editing is limited (Zhao et al., 2020). The zevoCDA1-SpRY-BE4max developed by Zhang et al. achieved NRN/NYN PAM compatibility by integrating the PAM-insensitive SpRY-Cas9 and the optimized CDA1 deaminase, and the editing efficiency at the GC site also increased to 42.67%, however by stander cytosines are often edited. (Zhang et al., 2024). In addition, Zheng et al. used zSpRY-ABE8e to break through the limitation of NGG PAM and achieve efficient A>G editing in the start codon (A_1_) of zebrafish tsr2 gene (Zheng et al., 2023). In practical applications, the problem of sequence context such as multiple Cs in the activity window may result in not just the intended conversion of the only 1 C, which is a significant challenge in accurate modeling. A narrow window can be an effective approach to resolve the problem. By removing the linker peptide and nuclear output signal (NES) from zevoCDA1-SpRY-BE4max, its variant zevoCDA1-198 narrows the window to C_1_∼C_5_, demonstrating exceptionally high precision when creating the ARS disease model, with the by stander editing rate below 0.23% and even reaching 0%, indicating almost no detectable non-target editing, and reduces the off-target effect from 36.07% to 6.04% (Zhang et al., 2024). Plus, Qin et al. generate a precise editor ABE-Umax-rest1 by introducing the N108Q mutation, which virtually eliminates bystander editing at other adenines with a narrower editing window of 1–2 nucleotides at A_5_ or A_6_ (Qin et al., 2024). These advances highlight the critical role of targeting window optimization for accurate genetic modeling of zebrafish.

Single-base editors enable precise base substitution without causing double-strand breaks (DSBs), significantly improving the purity of edits. Although ABE system demonstrated a product purity of up to 99.9% and a very low indels rate (<0.1%) (Gaudelli et al., 2017). The compatibility of editing systems may varies significantly among different species. For example, the original ABE7.10 was virtually inactive in zebrafish, and its variant, zABE7.10max, successfully achieved A>G after codon optimization and two-component nuclear localization signal (bis-bpNLS) modification G, but the indels rate was still 7.14∼22.20%, which was significantly higher than that in human cells (Qin et al., 2018b). In addition, ABE in zebrafish has a narrower editing window than in human cells, indicating that species-specific factors may affect the performance of editing tools (Qin et al., 2018b; Kim et al., 2017). Despite the challenges, zABE7.10 achieves high germline transmission (25∼58%) in zebrafish, providing a viable option for disease modeling (Qin et al., 2018b). In the future, strategies such as optimizing gRNA design and regulating chromatin accessibility should be further improved to further improve editing purity in zebrafish (Kim et al., 2017; Burger et al., 2016).

### 4.2 Off-target effects

Compared with traditional CRISPR-Cas9 nucleases, single-base editors significantly reduce chromosomal translocations, large deletions, and plasmid/retrotransposon insertion events by avoiding DSBs (Tao et al., 2023). However, the following off-target types still need to be of concern: gRNA-dependent DNA off-target, non-gRNA-dependent DNA off-target, and deaminase-mediated Cas-independent RNA off-target. The least costly way is to use CRISPOR (Concordet and Haeussler, 2018), Cas-OFFinder (Bae et al., 2014), etc., and other *in silico* simulations to predict off-target. Suitable for initial gRNA screening, but requires follow-up validation and is only suitable for gRNA-dependent DNA off-target. *In vitro* experiments can be used to detect off-target gRNA and non-gRNA-dependent DNA off-target using methods such as CIRCLE-Seq, which is highly sensitive and can cover the whole genome (Tsai et al., 2017), but still cannot reflect the real situation *in vivo*. Finally, it is suitable for all types of off-target by experiments *in vivo*. Cas-dependent DNA off-target can be detected using GUIDE-Seq and whole-genome sequencing (WGS), etc., while Cas-dependent RNA off-target can be detected using transcriptome analysis (Zuo et al., 2019; Grünewald et al., 2019). *In vivo* experiments can reflect the real biological environment, but due to the high cost and complex data analysis, they are not suitable for widespread use at present. For low-frequency off-target events (typically <1%), targeted deep sequencing, such as CIRCLE-seq, has become a more practical gold standard than WGS due to its high sensitivity and low cost.

In addition to off-target detection, a variety of optimization strategies have been developed to improve editing accuracy, including Cas9 variant integration, editor engineering, and delivery system optimization (Qin et al., 2018b; Burger et al., 2016; Li S. et al., 2021). Cas9 variant integration includes Cas9HiFi to reduce gRNA-dependent off-target by cutting interactions with non-target DNA-phosphate backbones (Slaymaker et al., 2016; Kleinstiver et al., 2016); in addition to Cas9HiFi, the double nicking strategy can improve specificity by mimicking DSBs with two adjacent nicks (Ran et al., 2013). Editor engineering includes codon optimization and NLS enhancement and replacement of nCas9 with dCas9. Codon optimization and nuclear localization signal enhancement can improve editing efficiency while reducing non-specific activity but may not eliminate gRNA-independent off-target, such as zABE7.10max (Qin et al., 2018b). Use dCas9 instead of nCas9 to reduce DNA nickase activity, which reduces the indel rate from 7.14∼22.20% to a lower level in zebrafish model, but at the expense of editing efficiency (Qin et al., 2018b). Optimization of delivery systems includes RNP delivery and co-selection strategies. The use of RNP delivery can further reduce the off-target rate (Burger et al., 2016); the co-selection strategy can simplify the off-target detection process by introducing phenotypic markers to screen individuals with high editing efficiency, but it relies on specific phenotypes and has a narrow scope of application (Li S. et al., 2021). Based on currently reported off-target analyses in zebrafish (Liang et al., 2022; Rosello et al., 2022; Zhang et al., 2024), single-base editing has not been associated with significant off-target effects. Moreover, even if off-target events do occur, they can be effectively mitigated in zebrafish through successive outcrossing.

The future path to balancing single-base editing accuracy and applicability may require the development of a combination of multi-omics analysis and novel BE variants (Zhao et al., 2021).

### 4.3 Addressing single nucleotide editing technologies

**FIGURE 5:**
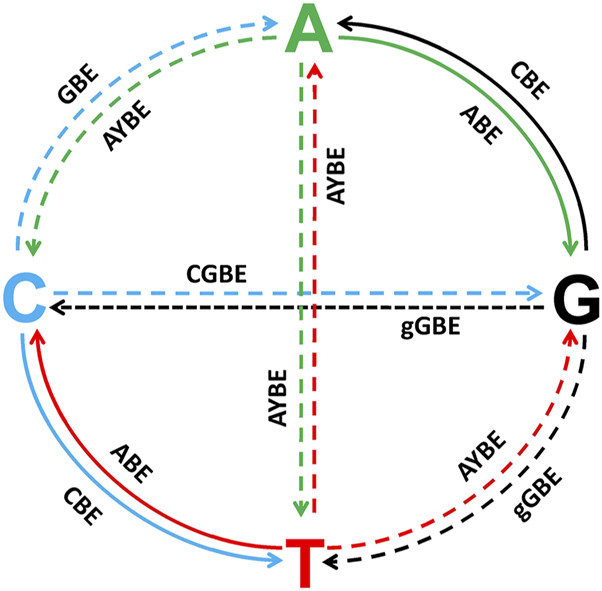
All the potential base substitutions performed by CRISPR-Cas9-derived base editing systems, with a focus on both achieved and unrealized editing capabilities in zebrafish. The four nucleotides (A, T, C, and G) are positioned at the ends of the cross-shaped diagram, and arrows indicate the directions of possible nucleotide conversions. Solid lines represent base substitutions that have already been successfully achieved using existing base editing technologies in zebrafish, while dashed lines indicate base substitutions that remain technically challenging or have not yet been achieved in zebrafish. Solid Lines (Achieved Substitutions): C→T, G→A: Achieved by cytosine base editors (CBEs), which deaminate cytosine into uracil, leading to a C•G to T•A transition after DNA repair. A→G, T→C: Achieved by adenine base editors (ABEs), which convert adenine to inosine, interpreted as guanine during DNA repair.Dashed Lines (Unrealized Substitutions): C→A (GBE), C→G (CGBE), G→C (gGBE), G→T (gGBE), A→T (AYBE), A→C (AYBE), T→A (AYBE), T→G (AYBE): These base substitutions remain technically challenging in zebrafish.

Derived from CRISPR-Cas9, base editing systems provide researchers with a versatile toolkit for single-nucleotide editing in zebrafish (Zhang et al., 2017; Liang et al., 2022; Zhang et al., 2024; Qin et al., 2024). However, these systems are limited in their ability to introduce all of the base substitutions required to model genetic disease in zebrafish (Kurt et al., 2021; Tong et al., 2023a; Chen et al., 2024) (Figure 5). Zhao et al. reported a glycosylase base editor (GBE) causing C-to-A transversions in *E. coli* and a C-to-G editor (CGBE) in mammalian cells, the core of which is substituting UGI with an uracil-DNA glycosylase (UNG) to excise the U base created by the cytidine deaminase, forming an apurinic/apyrimidinic (AP) site that initiates the DNA repair process, while using activation-induced cytidine deaminase (AID) in *Escherichia coli* and rat APOBEC1 in mammalian cells (Zhao et al., 2021). Chen et al. used the contrary principle of UNG-mediated base excision initiation to increase C-to-G transversions in cells, that is, the endogenous base excision repair (BER) pathway. By replacing UGI with BER proteins in CBE, they prevent UGI from inhibiting UNG and downstream BER, thereby enabling C:G to G:C base editors (CGBEs) (Chen et al., 2021). In addition, AYBE conducts A to C and A to T with the fusion of ABE8e and alkyladenine/3-methyladenine DNA glycosylase (AAG) (Tong et al., 2023a; Chen et al., 2024). Similarly, by fusing nCas9 with engineered AAG, a deaminase-free glycosylase-based guanine base editor (gGBE) allows G to C and G to T conversions (Tong et al., 2023b). Plus, to achieve C-to-T and A-to-G substitutions at the targeting site simultaneously, a novel fusion adenine and cytosine base editor (ACBE) has been generated by fusing a heterodimer of TadA and an AID to the N- and C-terminals of nCas9 respectively, showing promising prospect of the dual base editing (Xie et al., 2020). Currently, C to T and A to G transitions are relatively mature in zebrafish, however, other base substitutions such as C to A conducted by GBE (Zhao et al., 2021), C to G by CGBE (Chen et al., 2021), A to Y (A to C and A to T) by AYBE (Tong et al., 2023a; Chen et al., 2024), and G to Y (G to C and G to T) by gGBE (Tong et al., 2023b), have only been reported in cells.

In contrast, prime editing is capable of constructing almost any type of genetic mutation for zebrafish models of human disease (Li Y. et al., 2021). Prime editing technology was introduced by Anzalone et al. as a “search and replace” genome editing technique, able to introduce targeted insertions, deletions, and all 12 possible single-nucleotide base conversions, and their combinations in human cells without requiring DSBs or donor DNA templates. This system uses a special gRNA called pegRNA, which contains two parts: one is the guide sequence used to guide the binding of Cas9 protein to the target DNA, and the other is the RNA sequence containing the editing template. And unlike traditional Cas9, PE-guided editing uses Cas9-reverse transcriptase (Cas9-RT), a fusion protein that combines the functions of Cas9 protein and reverse transcriptase. Upon the Cas9 nicks the PAM-containing DNA strand, the prime editor then uses the newly liberated 3′ end at the target DNA site to prime reverse transcription using the extension in the pegRNA as a template, and finally achieves editing through the endogenous DNA repair mechanism (Anzalone et al., 2019). However, the application of prime editing in zebrafish is not so satisfying, with only about 30% precise editing efficiency obtained. (Qin et al., 2024; Petri et al., 2022; Ponnienselvan et al., 2023). Recently, HDR-based modification also comes into sight, as it can conduct G to C, A to C and G to A conversions, whereas the efficiency rages from 10% to 40% (Bai et al., 2020; Zhang et al., 2023; Carrington et al., 2022). By comparison, BEs have obvious advantages in versatility and editing efficiency. Therefore, in the future, the single base editing in zebrafish will still test the new base editor as the mainstream.

## 5 Conclusion

Single-nucleotide editing techniques have shown promising results in zebrafish, enabling studies into specific genes and their functions, and a better understanding of human physiological functions and processes (Tessadori et al., 2018). Human diseases caused by pathogenic single-nucleotide mutations can be modeled in zebrafish using base editing technologies, which produces similar phenotypes to those in mammalian models, like mice, while being more convenient and practical (Dalla Barba et al., 2023; Ikle et al., 2022). Base editing is featured with its broad applicability and prominently high efficiency in zebrafish compared with other single nucleotide editing technologies. Meanwhile, the high development efficiency promises a future of all kinds of base conversions. This review highlights that zebrafish will remain a preferred model organism for the foreseeable future. Furthermore, ongoing innovation and advancements in base editors will continue to drive the development of zebrafish models for studying human genetic diseases.
